# TiO_2_ eliminates *Hymenolepis nana* eggs via photocatalytic activity

**DOI:** 10.1371/journal.pntd.0013715

**Published:** 2025-11-10

**Authors:** Rong Mou, Hongyan Wang, Xuanyin Cui, Xiaomao Li, Yi Cheng, Wenting Chen, Xiujun Deng, Jin Chen, Ke Zhang

**Affiliations:** 1 Department of Parasitology, School of Basic Medicine, Guizhou Medical University, Guizhou Key Laboratory of Microbio and Infectious Disease Prevention & Control/ The Key and Characteristic Laboratory of Modern Pathogenicity Biology, Guiyang, China; 2 Department of Clinical Laboratory, Tongren People’s Hospital, Tongren, China; 3 College of Materials Science and Engineering, Xi’an University of Architecture and Technology, Xi’an, China; NEHU: North Eastern Hill University, INDIA

## Abstract

Titanium dioxide (TiO_2_) exhibits bactericidal, fungicidal, and virucidal effects under ultraviolet light irradiation. However, there are few reports on the photocatalytic effect of TiO_2_ against parasitic eggs. This study aims to preliminarily investigate the effect of TiO_2_ photocatalysis on the inactivation of *Hymenolepis nana* (*H*. *nana*) eggs. We employed the trypan blue staining method to assess the survival rate of 100 insect eggs, investigating the roles of light, TiO_2_ concentration, solution pH and light intensity in the process of inactivating eggs with TiO_2_. Morphological and structural damage to the eggs was observed using electron microscopy. The levels of reactive oxygen species (ROS) and adenosine triphosphate (ATP) within the eggs were measured using a fluorescent enzyme labeler, and the infectivity of TiO_2_-treated eggs was evaluated by oral-gavage in mice (8 mice per group). The results showed that under mercury lamp irradiation, with a TiO_2_ concentration of 1.0 mg/L, pH values ranging from 6 to 8, light intensity of 0.50 mW/cm^2^, and photocatalytic exposure for 2 h effectively inactivated *H*. *nana* eggs. Electron microscopy revealed that TiO_2_ photocatalysis caused eggs shrinkage and collapse of the spherical structure, along with the decrease number of mitochondria within the eggs. The TiO_2_ photocatalysis resulted in an increase in ROS content and a decrease in ATP content in eggs. These findings indicate that TiO_2_ photocatalysis disrupts the structural integrity and mitochondrial function of *H*. *nana* eggs by elevating ROS levels and depleting ATP, while simultaneously reducing the infection rate in mice to 12.5%. This study lays the groundwork for potential future applications of TiO_2_-based photoenergy-mediated inactivation of parasitic eggs in wastewater and drinking water treatment, ultimately benefiting public health.

## Author summary

This study preliminarily investigated the inactivation of *Hymenolepis nana* (*H*. *nana*) eggs using titanium dioxide (TiO_2_) photocatalysis. The trypan blue staining method was used to assess eggs viability under varying conditions of light, TiO_2_ concentration, pH, and light intensity. Under conditions of TiO_2_ concentration at 1.0 mg/L, pH between 6 and 8, and light intensity at 0.50 mW/cm^2^, optimal inactivation effects can be achieved after 2 h of exposure to mercury lamp irradiation. Electron microscopy revealed that photocatalytic treatment caused eggs shrinkage, collapse of the spherical structure, and a reduction in mitochondrial number. Furthermore, a significant increase in reactive oxygen species (ROS) and a decrease in adenosine triphosphate (ATP) levels were detected within the eggs. These findings suggest that TiO_2_ photocatalysis inactivates *H*. *nana* eggs by disrupting their structural integrity and mitochondrial function. This work lays a foundation for the potential application of TiO_2_-based technology to inactivate parasitic eggs in water treatment, offering promise for public health protection.

## 1. Introduction

*Hymenolepis nana* (*H*. *nana*) is a zoonotic parasitic worm that infects both humans and rodents, with adult worms primarily residing in the intestinal. Humans infection with *H*. *nana* usually cause hymenolepiasis. Mild infections of *H*. *nana* in humans have no obvious clinical symptoms, while severe infections manifest as abdominal pain, diarrhea, anemia, and fever [1,2]. *H*. *nana* has been classified by the World Health Organization (WHO) as a neglected zoonotic helminth [3], and it is endemic in regions such as Asia, Africa, Southern/ Eastern Europe, and Central/ South America [4]. It is estimated that 50–75 million people worldwide are infected [5,6]. The adult worms produce thousands of eggs, which are subsequently excreted into the external environment through feces [7]. Eggs that persist in untreated water, sewage, sludge, and wastewater are the primary sources of *H*. *nana* transmission [8,9]. Effective treatment of contaminated water sources is currently a key strategy for the prevention and control of infection. However, parasitic eggs possess highly resilient shells and structural components, rendering them resistant to commonly used disinfectants in wastewater treatment processes [10]. The remarkable resilience of *H*. *nana* eggs poses significant challenges for water treatment and wastewater management. Their robust outer shells confer exceptional resistance to conventional disinfection methods, enabling prolonged environmental survival and posing a major zoonotic threat.

Although sedimentation and solar disinfection are commonly used methods for treating sewage and fecal sludge, their effectiveness is limited. Parasite eggs can remain viable for extended periods in sedimentation tanks [11]. In addition, studies have shown that disinfectants used to treat parasitic eggs contamination in water can produce nitrogen-containing byproducts such as N-nitrosodiethylamine (NDEA), which are associated with carcinogenic and teratogenic effects [12]. Consequently, there is an urgent need to develop innovative and more efficient disinfection methods specifically targeting worm eggs.

Titanium dioxide (TiO_2_) is an n-type semiconductor due to the presence of oxygen vacancies, thus as electron donors in the electronic structure of TiO_2_ [13]. TiO_2_ is the most commonly used catalyst in the field of water purification [14]. The photocatalytic mechanism of TiO_2_ primarily involves the generation of electron-hole pairs upon excitation by ultraviolet light. These charge carriers react with water (H_2_O) or oxygen (O_2_) on the surface of the material to produce a large quantity of reactive oxygen species (ROS), such as hydroxyl radicals (•OH), superoxide anions (•O_2_⁻), and hydrogen peroxide (H_2_O_2_). These ROS exhibit toxicity toward microbial cell membranes, inhibit the formation of bacterial biofilms, and disrupt membrane integrity, thereby promoting oxidative damage to biomolecules such as proteins and ultimately leading to microbial death [13,15]. It has demonstrated strong inhibitory effects against *Escherichia coli* (*E*. *coli*), *Pseudomonas aeruginosa* (*P*. *aeruginosa*), *Staphylococcus aureus* (*S*. *aureus*), and *Saccharomyces cerevisiae* (*S*. *cerevisiae*) [16,17], and can also effectively inactivate *Candida albicans* in water [18]. In addition to degrading microorganisms in water, this material can also reduce the activity of viral envelope proteins and inactivate viruses when exposed to natural light, thereby preventing viral transmission [19,20]. Some studies also find that other nanoparticle composites (g-C_3_N_4_/Fe_2_O_3_, MWCNTs-CuNiFe_2_O_4_, Fe_2_O_3_/bentonite/TiO_2_, and MMT/CuFe_2_O_4_) exhibit certain degradation effects on ampicillin (AMP), ciprofloxacin (CIP), and amoxicillin (AMX) solutions in water through ROS [21–24]. However, there have few reports on the lethality of TiO_2_ against parasitic eggs, and the mechanisms by which it may exert such effects remain unclear. Unlike TiO_2_ and other nanomaterials widely reported in the literature for degrading organic pollutants, we shift our focus from degrading chemical molecules or microorganisms to inactivating highly environmentally resistant parasite eggs. This provides a novel strategy for TiO_2_ to effectively treat parasite eggs that are difficult to eliminate with conventional disinfectants.

In addition, the (001) crystal plane pure TiO_2_ nanosheets employed in this study offered advantages of low cost and simple preparation compared to complex composite materials. They also exhibit superior catalytic performance and adsorption capacity relative to the (101) facet of anatase TiO_2_, achieving excellent results at lower catalyst concentrations and light intensities [25]. This indicates greater application potential and environmental friendliness in real-world water treatment scenarios. In this study, the ovicidal effect of TiO_2_ on *H*. *nana* eggs through photocatalysis under mercury lamp irradiation was determined. This finding confirmed the significant ovicidal efficacy of TiO_2_, elucidating that ROS generated by TiO_2_ disrupted the structural integrity of insect eggs and played a crucial role by inducing mitochondrial dysfunction. This provides foundational data for further understanding the photocatalytic inactivation mechanisms of TiO_2_ against other parasitic eggs present in water.

## 2. Materials and methods

### 2.1. Ethics statement

All animal experiments of the current study were approved by the Animal Ethics Committee of Guizhou Medical University (approval No. 2100346).

### 2.2. Reagents

The 0.4% (v/v) trypan blue staining solution (BL627A) and 2.5% (v/v) electron microscopy fixative (BL911A) were purchased from Biosharp (Anhui, China); 5% (v/v) NaClO standard solution was obtained from Shenzhen Fulin Instrument Technology Co., Ltd. (Shenzhen, China); ATP Assay Kit (S0026) and ROS Assay Kit (S0033S) were offered by Beyotime (Shanghai, China). The 812 embedding resin (90529-77-4) and osmium tetroxide (18456) were obtained from Servicebio (Wuhan, China).

TiO_2_ used in this study is a TiO_2_ nanosheet with excellent (001) exposed facets that were synthesized by a solvothermal method with a diameter of about 220 nm. It was characterized and validated by our group with scientific literatures published elsewhere [26–28].

### 2.3. Assessing the survival rate of *H*. *nana* eggs by trypan blue staining

Parasitic worms were obtained from hamsters purchased at a pet market in Nanming District, Guiyang City, China. Morphological and molecular biological identification were performed to identify the parasites as *H*. *nana* [29–31]. Gravid proglottids of adult *H*. *nana* were minced using sterilized surgical scissors to release the eggs. An equal volume of eggs suspension and 0.4% (v/v) trypan blue solution was mixed at a 1:1 ratio, gently homogenized, and store at room temperature for 5 min. 100 eggs were observed and counted under a microscope and the survival rate was calculated. Viable eggs contained unstained oncospheres, while non-viable eggs exhibited blue-stained oncospheres (S1 Fig).

### 2.4. Establishment of the TiO_2_ photocatalytic reaction system

**Fig 1:**
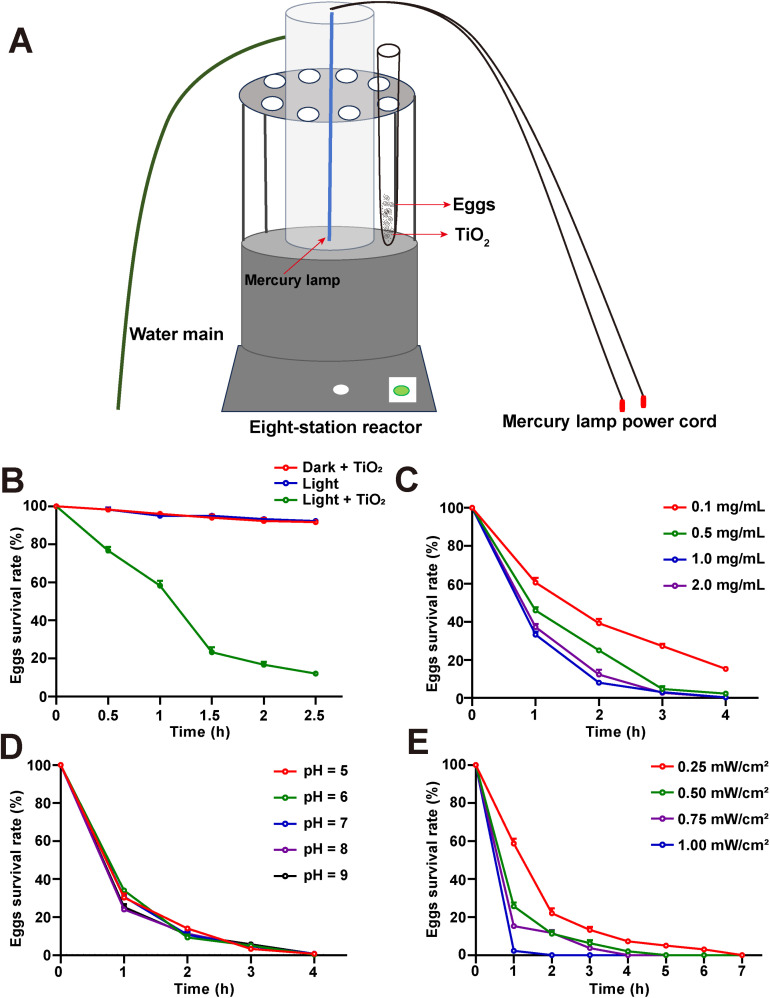
1.0 mg/mL TiO_2_ can effectively eliminate *H*. *nana* eggs under light exposure. (**A**) Experimental design diagram. A test tube containing a mixture of eggs and TiO_2_ was placed in an Eight-station reactor, and a magnetic stirrer was activated to ensure full contact between TiO_2_ and the eggs, with a mercury lamp in the center, and a quartz tube outside the mercury lamp that could be connected to a water main to provide thermal insulation. (**B**) The effect of light/ dark conditions on the *H*. *nana* egg survival rate (%) by 0.5 mg/mL TiO_2_ with 0.50 mW/cm² light intensity and pH 7.0. (**C**) The *H*. *nana* egg survival rate (%) at different concentrated TiO_2_ with 0.50 mW/cm² light intensity and pH 7.0. (**D**) The effect of different pH values on the *H*. *nana* egg survival rate (%) by 1.0 mg/mL TiO_2_ with 0.50 mW/cm² light intensity. (**E**) The effect of different mercury lamp light intensities on the *H*. *nana* egg survival rate (%) by 1.0 mg/mL TiO_2_ with pH 7.0. Data are presented as mean  +  SD in (B-E), n  =  3 per group for (B-E).

TiO_2_ (001) powder was placed in a 1.5 mL EP tube containing 1 mL ddH_2_O solvent. The powder was dispersed using an ultrasonic cell disruptor and a shaker for 30 min. The mixture was transferred to a 10 mL sterilized glass test tube. 250 μL of eggs suspension with a total number of 12,500 eggs was added, followed by additional ddH_2_O solvent to bring the volume to 5 mL, forming a suspension of TiO_2_ eggs at a specific concentration. The test tube containing the TiO_2_/ eggs suspension was placed in an Eight-station reactor (Yihui Analytical Instruments Co., Ltd.), where an external circulating water system insulated the hollow quartz tube. A high-pressure mercury lamp was then turned on and the light intensity was adjusted. The temperature-controlled magnetic stirrer of the reactor was set to stir at 120 rpm to ensure full contact between the TiO_2_ and the eggs (Fig 1A). The TiO_2_/ eggs suspension was collected according to the experimental time.

### 2.5. Inactivation effects of TiO_2_ on *H*. *nana* eggs under different conditions (light and dark, different TiO_2_ concentrations, different pH, and different light intensities)

For light and dark conditions: a suspension of 5 mL of 0.5 mg/mL TiO_2_ and 2,500/mL egg was prepared. The TiO_2_/ eggs suspension was placed in an Eight-station reactor and the light intensity was adjusted to 0.25 mW/cm^2^ to set up a photocatalytic reaction system. Meanwhile, a dark control group (dark + TiO_2_) and a mercury light irradiation group without TiO_2_ (light) were set up. For different TiO_2_ concentrations: a suspension containing 12,500 eggs were prepared in a final reaction volume of 5 mL with TiO_2_ concentrations of 0.1 mg/mL, 0.5 mg/mL, 1.0 mg/mL and 2.0 mg/mL. The TiO_2_/ eggs suspension was placed in an Eight-station reactor and the light intensity was set to 0.25 mW/cm^2^. For different pH values: use solvents with pH values of 5, 6, 7, 8, and 9 to prepare suspensions with a final volume of 5 mL, a TiO_2_ concentration of 1.0 mg/mL, and a concentration of 2,500 eggs/mL. Place the TiO_2_/ eggs suspension into an octuplex reactor and set the light intensity to 0.25 mW/cm^2^. For different light intensities: double distilled water was used as the reaction suspension with a TiO_2_ concentration of 1.0 mg/mL and an egg concentration of 2,500 eggs/mL. A mercury lamp was adjusted to light intensities of 0.25 mW/cm^2^, 0.50 mW/cm^2^, 0.75 mW/cm^2^, and 1.00 mW/cm^2^ and the TiO_2_/ eggs suspension was placed in an Eight-station reactor.

Samples were taken at different reaction timepoints. After trypan blue staining, the samples were observed under the microscope, and the number of viable eggs out of 100 counted eggs was recorded to calculate the egg survival rate.

### 2.6. Hatching experiment of *H. nana* eggs after TiO_2_ photocatalysis

The optimal incubation concentration of NaClO is 0.6% (v/v), and the detailed steps are described in the S1 Text. A suspension of 100 μL of 0.5 mg/mL TiO_2_ and 2,500/mL egg was photocatalyzed under a mercury lamp with a light intensity of 0.50 mW/cm^2^ for 2 h. Then, 400 μL of 0.6% (v/v) NaClO solution was added. Mix well and let stand for 3–5 min at room temperature. The reaction was stopped by the addition of 10 mL of 0.85% (v/v) NaCl solution. The supernatant was centrifuged at 865 x g for 5 min, and the precipitate was washed twice with 0.85% (w/v) NaCl solution. The precipitate was resuspended with 200 μL of 0.85% (w/v) NaCl solution and 100 eggs were observed and counted under a microscope to calculate the hatching rate of the eggs.

### 2.7. Scanning electron microscope (SEM) and transmission electron microscope (TEM) observations of *H*. *nana* eggs damage

A suspension containing 12,500 eggs with PBS was treated with 0.6% (v/v) NaClO, and 1.0 mg/mL TiO_2_ after 2 h and 4 h of photocatalysis with the light intensity of 0.50 mW/cm^2^ respectively. The suspension was centrifuged at 865 x g for 5 min, the egg precipitates were collected and fixed with 2.5% (v/v) electron microscope fixative and stored at 4°C. For SEM: use a JSM-IT700HR scanning electron microscope (JEOL, Japan) to acquire images of the samples. For TEM: use an HT7800/HT770 transmission electron microscope (Hitachi, Japan) to acquire images of the samples.

Specific procedures for SEM and TEM are described in the “S1 Text” section.

### 2.8. Detection of ROS and ATP in *H*. *nana* eggs

A suspension containing 12,500 eggs was taken and treated with PBS, 0.6% (v/v) NaClO, and 1.0 mg/mL TiO_2_ after 2 h of photocatalysis with the light intensity of 0.50 mW/cm^2^. The suspension was centrifuged at 865 x g for 5 min, the supernatant was discarded and the precipitate was retained. For ROS: Mean Fluorescence Intensity (MFI) was measured at 485/ 530 nm excitation/ emission wavelengths using a SYNERGY-H4 multipurpose microplate reader (Bio-Tek, USA). After sealing the slides, used an inverted fluorescence microscope (Eclipse 80i, Nikon Ltd, Japan) to capture images. For ATP: Relative Light Unit (RLU) was measured at 485/ 530 nm excitation/ emission wavelengths using a SYNERGY-H4 multipurpose microplate reader (Bio-Tek, USA).

Specific procedures are described in the “S1 Text” section.

### 2.9. ICR mouse experiment

Healthy female ICR mice (6–8 weeks old, 20 ± 2 g) were obtained from the Experimental Animal Center of Guizhou Medical University (SCXK (Jing) 2019–0010). Animals were kept under specific pathogen-free (SPF) conditions at room temperature of 20–22°C, and subjected to a controlled 12-h light/ dark cycle. All mice were given adaptive feeding for at least one week before formal experiments. Mice were randomly divided into 3 groups: infection control group (*H*. *nana*), 0.6% (v/v) NaClO treatment group (NaClO), and 1.0 mg/mL TiO_2_ treatment group (TiO_2_) after 2 h of photocatalysis with light intensity of 0.50 mW/cm^2^. The 500 *H*. *nana* eggs were orally administered per mouse. The body weight of each mouse was measured and recorded from the day of gavage to day 14 post-gavage.

The number of eggs per gram of feces (EPG) was calculated using the McMaster counting chamber as follows:

EPG = [(n1 + n2)/2] × [10/(0.15 x n3)], where (n1 + n2) is the total number of eggs and n3 is the weight of the feces [32].

Additionally, the mice were euthanized under anesthesia and dissected at Day 14. The number and length of the adult worms in the intestinal lumen were counted. The number of eggs, adults, and adult length in mice not infected with *H*. *nana* were defaulted to zero.

### 2.10. Statistical analysis

The statistical analysis and graphical representation were performed using GraphPad Prism (Version 5.0). The data were presented as mean + SD. Measurements were first subjected to normality tests, and the Homogeneity of Variance Test was performed between groups. Mann-Whitney U-test was employed as a non-parametric statistical method to analyze data that deviated from normal distribution. One-way analysis of variance (ANOVA) was used to test differences between multiple groups, and the Mann-Whitney U-test and t-test were used to compare differences between two groups. The *p* value less than 0.05 means a statistically significant difference; ns means no statistically significant.

## 3. Results

### 3.1. Experimental design and the killing effect of TiO_2_ on *H*. *nana* eggs

**Fig 2:**
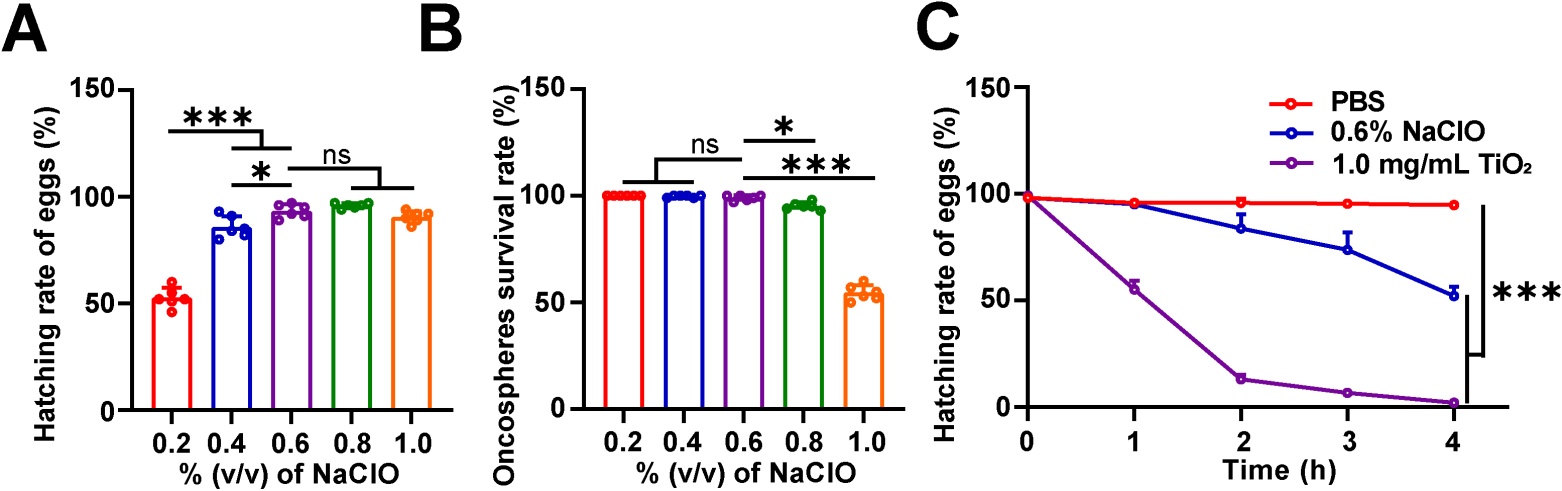
The effect of NaClO and TiO_2_ on the hatching parameters of *H*. *nana* eggs. **(A)** Hatching rate (%) of *H*. *nana* eggs. **(B)** Survival rate (%) of oncospheres after hatching from *H*. *nana* eggs. **(C)** Hatching rate (%) of *H*. *nana* eggs after TiO_2_ treatment. Data are presented as mean + SD in **(A-C)**, *n* = 6 per group for (A) and **(B)**, *n* = 3 per group for **(C)**, * *p* < 0.05, *** *p* < 0.001, ns: not statistically significant.

To explore the killing effect of TiO_2_ on *H*. *nana* eggs, the following methods were used (Fig 1A). Eggs were exposed to TiO_2_ under dark conditions (Dark + TiO_2_) and under mercury lamp irradiation without TiO_2_ (Light). No significant change in eggs survival rate was observed. However, when TiO_2_ photocatalysis (Light + TiO_2_) was applied for 2.5 h, the egg survival rate dramatically decreased to 12%, showing a significant decline, while the survival rates of eggs in the Dark + TiO_2_ and Light groups remained over 90% (Fig 2B). These results indicated that TiO_2_ required light exposure to inactivate the eggs. At a TiO_2_ concentration of 1.0 mg/mL, the egg mortality rate was higher than at 0.1 mg/mL, and the rate of mortality was also faster. The TiO_2_ concentration was increased from 1.0 mg/mL to 2.0 mg/mL did not increase the mortality rate of the eggs (Fig 2C). When eggs were exposed to solvent pH values of 5, 6, 7, 8, and 9 for 1 h, the highest eggs mortality (average of 74.67%) was observed at a high pH value of 9. After 2 h of exposure, the lowest mortality (average of 86%) was observed at pH 5. However, when the exposure time exceeded 2 h, no significant effect of different pH values on eggs inactivation was observed. These results indicated that within the pH range of 6–8, changes in pH did not significantly affect egg inactivation (Fig 2D). As the light intensity increased from 0.25 mW/cm^2^ to 1.00 mW/cm^2^, the time required for complete egg death decreased. During 0–3 h of light exposure, egg survival decreased significantly with increasing light intensity, especially at 1 h of light exposure. With the increase of irradiation time, there was no significant difference in egg mortality between light intensity of 0.50 mW/cm^2^ and 0.75 mW/cm^2^ after 2–3 h (Fig 2E). The above results demonstrate that under visible light exposure, TiO_2_ at a concentration of 1.0 mg/L, with a light intensity of 0.50 mW/cm^2^, and a pH range of 6–8, effectively inactivates *H*. *nana* eggs after 2 h of photocatalytic treatment.

### 3.2. TiO_2_ photocatalysis reduces the hatching ability of *H*. *nana* eggs

To determine the optimal NaClO concentration for hatching *H*. *nana* eggs, we first evaluated the hatching rate and the survival rate of the oncospheres after hatching from fresh *H*. *nana* eggs treated with NaClO. The results showed that compared to the hatching rate at 0.6% v/v NaClO, there was no significant difference in the rates at 0.8% v/v and 1.0% v/v NaClO, while the hatching rates were reduced at 0.2% v/v and 0.4% v/v NaClO (Fig 2A). Similarly, compared to the survival rate of oncospheres at 0.6% v/v NaClO, there was no significant change in survival rates at 0.2% v/v to 0.6% v/v NaClO, whereas the survival rate of oncospheres decreased at 0.8% v/v and 1.0% v/v NaClO, with egg dissolution observed at 1.0% v/v NaClO (Fig 2B). These results indicated that the 0.6% v/v NaClO was optimal for hatching *H*. *nana* eggs, and therefore, 0.6% v/v NaClO was used for hatching experiments on *H*. *nana* eggs after photocatalysis. Comparing to the PBS and NaClO groups, the hatching rate of eggs subjected to TiO_2_ photocatalysis and then incubated with NaClO was significantly lower, with the rate dropping to below 15% after 2 h treatment (Fig 2C). The results indicate that TiO_2_ photocatalysis can reduce the hatching ability of *H*. *nana* eggs.

### 3.3. SEM observation of damage to eggs

**Fig 3:**
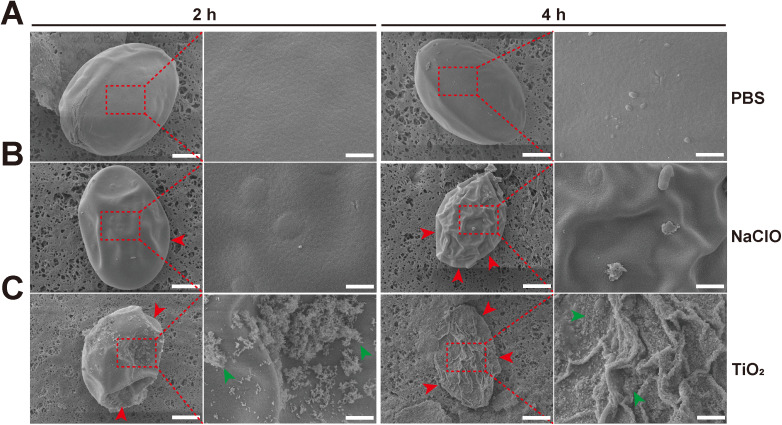
Representative SEM images of *H*. *nana* eggs at 2 h and 4 h timepoints. **(A)** PBS treatment group. (**B**) 0.6% (v/v) NaClO treatment group. (**C**) 1.0 mg/mL TiO_2_ with 0.50 mW/cm^2^ light intensity treatment group. The red arrowheads indicate surface depressions, and the green arrowheads point to TiO_2_. For the 2 h and 4 h timepoints, the scale bars on the left panel = 10 μm, and the scale bars on the right panel = 1 μm.

To observe the damage to the surface ultrastructure of *H*. *nana* eggs caused by TiO_2_ photocatalysis, SEM was used to examine eggs treated with PBS, NaClO, and TiO_2_ photocatalysis for 2 h and 4 h, respectively. The results showed that PBS-treated eggs were oval-shaped, plump, with smooth surfaces and no obvious changes. There was no significant difference in eggs treated with PBS for 2 h and 4 h (Fig 3A). In contrast to PBS-treated eggs, those treated with NaClO for 2 h had a relatively regular shape, but the surface was not smooth, showing micro-depressions. After 4 h of NaClO treatment, the eggs exhibited an irregular shape with noticeable shrinkage (Fig 3B). TiO_2_-treated eggs for 2 h showed a reduced size, almost losing their spherical shape, with a rough surface and noticeable depressions. A large amount of TiO_2_ was adsorbed on the surface of the eggs. After 4 h of TiO_2_ treatment, the eggs were even smaller, showing obvious degeneration, shrinkage, and had completely lost their spherical shape. The surface exhibited extensive folding, and a large amount of TiO_2_ material was adsorbed on the surface (Fig 3C). These results indicate that both NaClO and TiO_2_ photocatalysis can cause damage to *H*. *nana* eggs, with TiO_2_ showing more noticeable damage.

### 3.4. TEM observation of eggs damage

**Fig 4:**
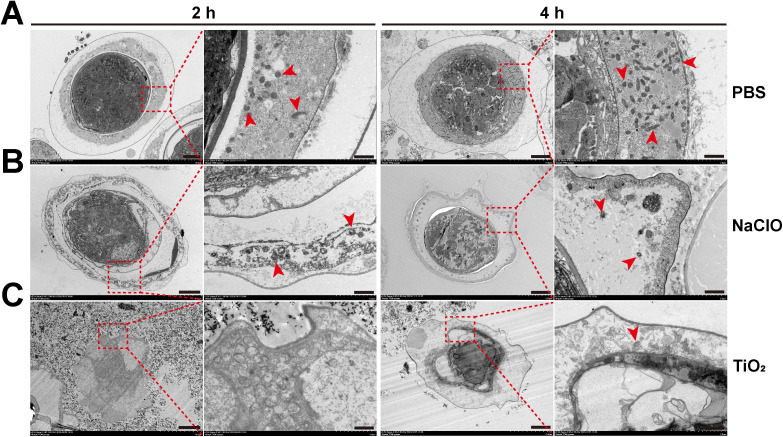
Representative TEM images of *H*. *nana* eggs morphology and mitochondria at 2 h and 4 h timepoints. **(A)** PBS treatment group. (**B**) 0.6% (v/v) NaClO treatment group. (**C**) 1.0 mg/mL TiO_2_ with 0.50 mW/cm^2^ light intensity treatment group. The red arrowheads indicate mitochondria. For the 2 h and 4 h timepoints, the scale bars on the left panel = 5 μm, and the scale bars on the right panel = 1 μm.

To further investigate the damage to egg ultrastructure by TiO_2_ photocatalysis, TEM was used to observe eggs treated with PBS, NaClO, and TiO_2_ photocatalysis for 2 h and 4 h. The results showed the PBS-treated group after 2 h and 4 h, the egg structure remained relatively intact, with the hexacanth embryo intact and mitochondria within the embryo membrane being numerous and well-preserved (Fig 4A). In the NaClO-treated groups, after 2 h and 4 h, some damage was observed, with slight shrinkage; the hexacanth embryo remained largely intact, but the number of mitochondria in the egg membrane was reduced (Fig 4B). In the TiO_2_-treated groups after 2 h and 4 h, the damage was more severe, with significant shrinkage, a clear loss of the hexacanth embryo structure, and very few mitochondria within the egg membrane. No TiO_2_ was visible inside the eggs (Fig 4C). These results indicate that both NaClO and TiO_2_ can damage eggs and reduce mitochondrial quantity, but TiO_2_ causes more severe damage to the eggs compared to NaClO.

### 3.5. Detection of mitochondrial function-related indicators in eggs

**Fig 5:**
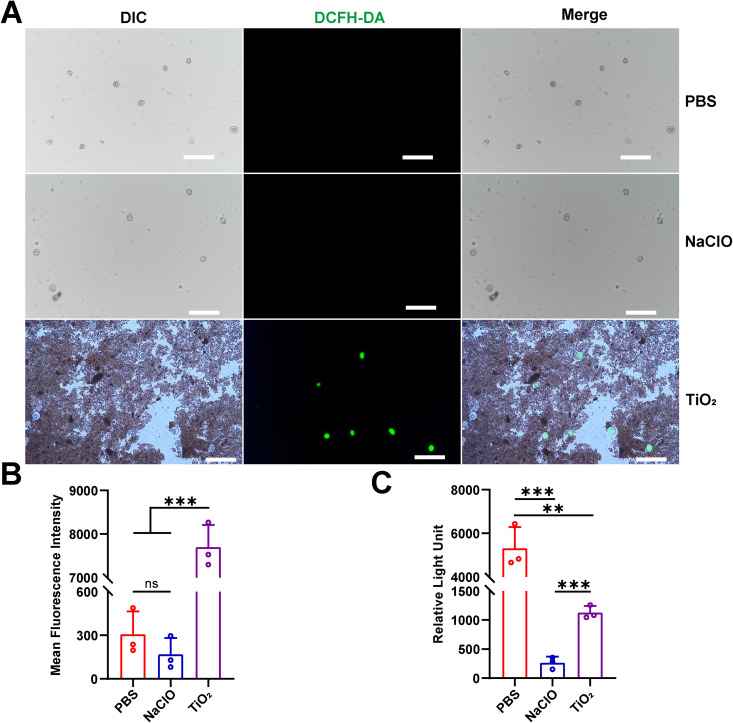
Effects of TiO_2_ photocatalysis on ROS and ATP levels in eggs. **(A)** Microscopic observation of the binding of DCFH-DA dye to the eggs (scale bars = 200 μm). **(B)** Fluorescent enzyme labeler detection of the Mean Fluorescence Intensity (MFI) of DCFH-DA positive eggs, and MFI represents ROS levels. **(C)** Fluorescent enzyme labeler detection of the RLU in the eggs, and RLU represents ATP levels. Data are presented as mean + SD in (B) and **(C)**, *n* = 3 per group for (B) and **(C)**, ** *p* < 0.01, *** *p* < 0.001, ns: not statistically significant.

Mitochondrial abnormalities of eggs after TiO_2_ photocatalysis was showed via TEM, since ROS are primarily produced by mitochondria, we used the DCFH-DA green fluorescent probe to detect the ROS content inside the eggs. This dye binds with ROS inside the eggs and emits fluorescence, allowing to assess the ROS levels using Mean Fluorescence Intensity (MFI). Under the microscope, we observed that the ROS content in the TiO_2_ photocatalysis group was significantly higher than in the PBS and NaClO treatment groups (Fig 5A). Fluorescent enzyme labeler measurements also showed that the MFI in the TiO_2_ photocatalysis group was extremely higher, indicating increased ROS levels in the eggs compared to the PBS and NaClO groups (Fig 5B). These results suggest that TiO_2_ photocatalysis leads to the accumulation of ROS in the eggs. Mitochondria also produce ATP, which provides the energy required for cellular functions. To further examine the ATP content in eggs after photocatalysis, we used an ATP assay kit to measure ATP levels. The results showed that the Relative Light Unit (RLU) in the TiO_2_ photocatalysis group was significantly lower than in the PBS group, but remarkably higher than in the NaClO group (Fig 5C). These results indicate that TiO_2_ photocatalysis impairs mitochondrial function of eggs.

### 3.6. Significant decrease in infection rate of mice infected with TiO_2_-treated eggs

**Fig 6:**
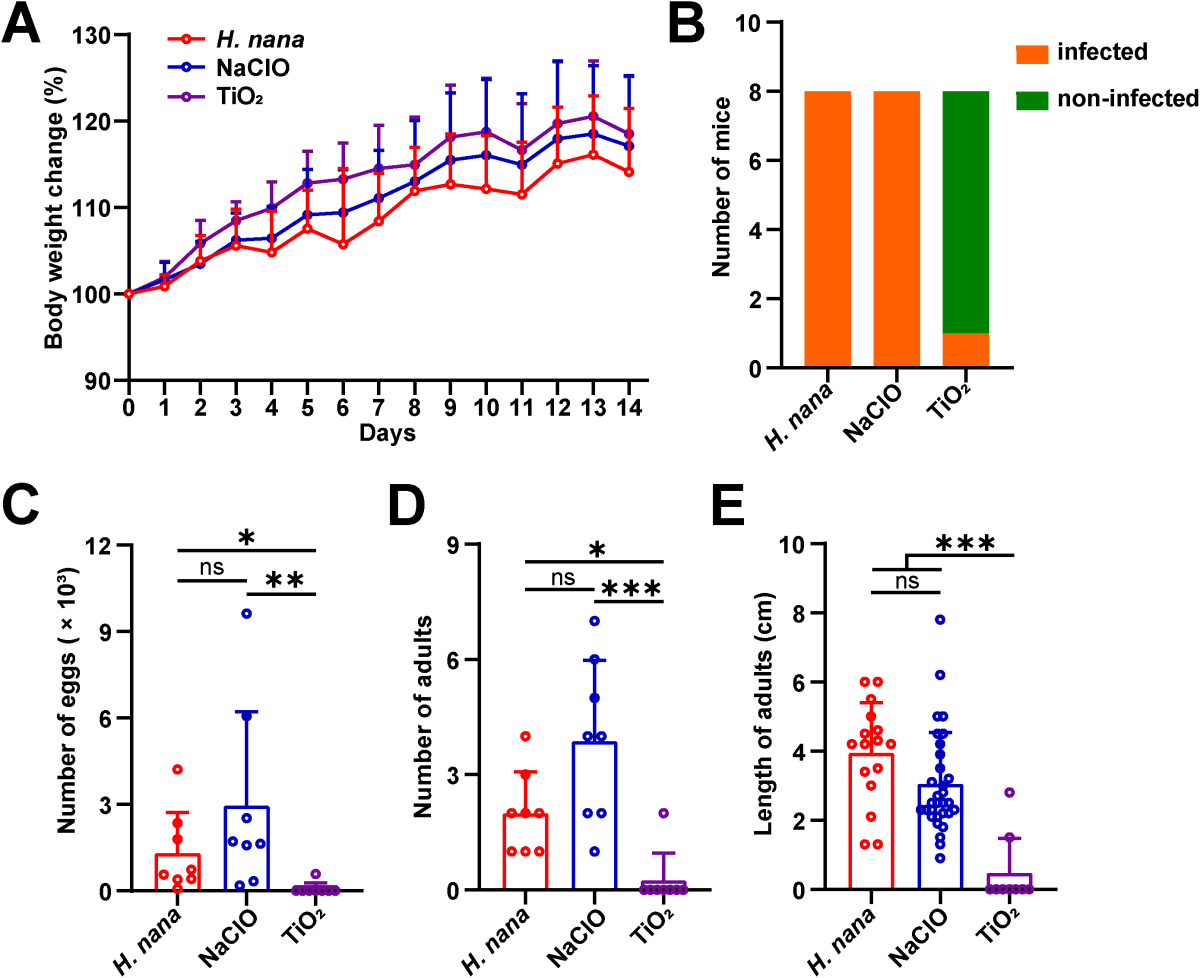
Changes in mouse body weight and *H*. *nana* infection load. **(A)** Body weight changes: the initial body weight was set as 100%. **(B)** The height of the orange bar indicates the number of infected mice and the height of the green bar indicates the number of non-infected mice. **(C)** Number of eggs detected in mouse feces. **(D)** Number of adults dissected into the intestinal lumen of each mouse. **(E)** Length of adults. Data are presented as mean + SD in (A, C-E), *n* = 8 per group for **(A-D)**, *n* = 9, 16, and 31 per group for **(E)**. * *p* < 0.05, ** *p* < 0.01, *** *p* < 0.001, ns: not statistically significant.

To further verify whether the eggs treated with TiO_2_ photocatalysis still have infectivity, we gavaged mice with TiO_2_-treated eggs. The results showed no statistically significant difference in body weight between the groups at the respective timepoints (Fig 6A). However, *H*. *nana* eggs were detected in the feces of mice from the *H*. *nana* and NaClO groups, with an infection rate of 100% (8/ 8). Notably, in the TiO_2_-treated group, only one mouse’s feces tested positive for *H*. *nana* eggs, with an infection rate of 12.5% (1/ 8) (Fig 6B). Compared with the *H*. *nana* group, the NaClO-treated group showed an increasing trend in the number of eggs and adults, but it was not statistically significant. Dramatically, the number of eggs, the number of adults dissected from the intestinal lumen of mouse, and the length of *H*. *nana* adults in the TiO_2_-treated group were significantly less than those in the *H*. *nana* and NaClO groups. (Fig 6C-6E). These results indicate a decrease in the infectivity of the *H*. *nana* eggs post TiO_2_ treatment.

## 4. Discussion

*H*. *nana* infection is primarily transmitted through the fecal-oral route, eggs are commonly found in raw water, sewage, sludge, and wastewater, serving as the main source of infection for *H*. *nana*. TiO_2_ is a novel nanomaterial, owns a range of advantages such as being non-toxic, supreme chemical stable, and cost-affordable [33]. It is widely used in wastewater treatment, environmental decontamination, and other fields [34–36]. The photocatalytic activity of TiO_2_ is directly related to its exposed crystalline facets [37]. The (101) facet of anatase TiO_2_ is thermodynamically stable, but its energy density is only 0.44 J/m^2^, which is deficient in absorbing solar energy. The energy density of the TiO_2_ (001) facet is practically twice as high as that of the (101) facet (0.90 J/m^2^), and the catalytic properties and adsorption of TiO_2_ improve with the increase in the percentage of the area of the (001) facet [25]. Therefore, the catalytic properties and adsorption of TiO_2_ (001) we used will be better compared to that of TiO_2_ of anatase type (101). TiO_2_ has been found to be lethal to fungi, bacteria, viruses, and insects [16,20,38]. It is still unknown whether TiO_2_ is lethal to parasite eggs. In this study, it was found that TiO_2_ could inactivate *H*. *nana* eggs by destroying the structure and mitochondrial function of the eggs through photocatalytic activity. This study represented the first systematic evaluation of TiO_2_ photocatalytic activity against worm eggs. Furthermore, we transcended the limitations of survival rate assessment alone by visualizing ultrastructural damage via electron microscopy and quantitatively detecting key biochemical indicators (ROS and ATP) to validate mitochondrial dysfunction. Finally, we innovatively linked in vitro findings to a validated in vivo infection model, providing compelling evidence for the practical application potential of this method.

TiO_2_ photocatalytic inactivation of pathogens in water depends on factors such as light intensity and the type of pathogen [39]. In this study, we found that the inactivation effect of TiO_2_ on *H*. *nana* eggs is mainly affected by factors such as light exposure, solution pH, material concentration, and light intensity. TiO_2_ did not show any lethal effect on the eggs under dark condition, and the best inactivation effect was observed at a TiO_2_ concentration of 1.0 mg/mL. These results suggest that TiO_2_ inactivates parasitic eggs only under certain conditions, specifically requiring light exposure for the inactivation.

The hatching rate of *H*. *nana* eggs significantly decreased after TiO_2_ photocatalysis, with the hatching rate being much lower than that of NaClO after 2 h of treatment. SEM results showed that TiO_2_ photocatalysis caused morphological and structural damage to the eggs, such as shrinkage, deformation, and loss of spherical shape. The mitochondria and other organelles inside the eggs were swollen and dissolved, and the substance structure of the hexacanth embryos disappeared. These findings indicate that although the eggs treated with photocatalysis maintained some morphological features, they were essentially dead and lost their infectious characteristics. SEM results revealed TiO_2_ adsorption on the insect egg surface, while TEM observations showed that TiO_2_ itself does not penetrate the eggshell to directly disrupt its structure. Since the generated ROS and free radicals undergo redox reactions on the photocatalyst surface. We therefore hypothesize that the sustained ROS produced during photocatalysis damage cellular organelles such as mitochondria in the eggs, ultimately leading to the death of oocyte.

Mitochondria are the energy centers of cells, generating large amounts of ATP to provide energy to the cell, as well as being the primary sites for the production of ROS. Excessive and uncontrolled ROS production can harm the cell, causing mitochondrial and tissue damage, reducing ATP levels, and ultimately leading to cell death [40,41]. Studies have shown that TiO_2_ produces large amounts of ROS when excited by visible light, thus destroying bacteria [13]. After *Foraminifera* are exposed to TiO_2_ for 1 h, excessive ROS leads to mitochondrial membrane depolarization and genotoxicity [42]. Additionally, the ROS generated within parasites and changes in mitochondrial membrane potential are the primary causes of *Leishmania* parasite death [43]. TEM observations revealed that both NaClO and TiO_2_ treatments reduced the number of mitochondria in the eggs. This morphological disruption strongly correlated with functional testing outcomes: a significant increase in ROS levels and a sharp decline in ATP content. We hypothesized that ROS generated by TiO_2_ initially attacked and disrupted mitochondrial membrane structures, leading to reduced numbers and functional loss. As cellular powerhouses, the collapse of mitochondrial function directly disrupted ATP synthesis. This vicious cycle of morphological and functional deterioration ultimately resulted in egg inactivation. Although our study confirmed elevated ROS and reduced ATP levels, the specific mechanisms by which ROS damage mitochondria and egg structures remain unclear. Future work necessitates the use of chemical probes to precisely attribute the degradation of *H. nana* eggs to a particular active species. This will be accomplished by the free radical trapping experiment that introduces ammonium oxalate to quench holes (H^+^), tert-butanol to quench hydroxyl radicals (•OH), and p-benzoquinone to quench superoxide radicals (•O_2_^-^) [28].

Mouse infection experiments confirmed the biological significance of the aforementioned in vitro findings. Although TiO_2_ treatment did not achieve 100% absolute inactivation (one infection still occurred), it reduced the infection rate from 100% in the control group to 12.5%. In the sole infected mouse, the number and length of adult worms recovered from the intestine were also significantly reduced. This observation indicates that even if a few eggs survived, their ability to mature and reproduce was severely impaired. These results suggest that TiO_2_ -treated eggs have a reduced infectivity to the host. Current traditional disinfection methods have issues such as secondary pollution, toxicity, resistance, and irritant side effects [44], and NaClO used for treating parasitic eggs in water can lead to carcinogenic, teratogenic, and other side effects [12]. Additionally, sedimentation treatment and solar disinfection are not very effective in treating parasitic eggs in wastewater and fecal sludge [45]. Notably, although NaClO treatment also reduced egg hatching rates and caused structural damage, it showed no statistically significant difference from the control group in reducing mouse infection rates. This contrasts sharply with the results from the TiO_2_ treatment group. We postulated that this discrepancy stems from their differing mechanisms of action: NaClO as a potent oxidizing agent, likely primarily targets the outer shell of parasite eggs, whereas ROS generated by TiO_2_ photocatalysis could more effectively penetrate and disrupt key internal organelles within the eggs, such as mitochondria. Therefore, TiO_2_ photocatalysis technology holds promise as an alternative method to conventional disinfection treatments for parasitic eggs.

The experiment was conducted under idealized laboratory conditions. Whether organic matter, turbidity, and complex microbial communities present in real aquatic environments (such as wastewater) significantly interfere with the photocatalytic efficiency of TiO_2_ warrants further investigation. In subsequent studies, the inactivation efficiency of TiO_2_ should be tested in real wastewater, and methods to enhance its activity and stability in complex environments through optimization should be explored. Concurrently, research should be extended to other parasite eggs (such as *Trichuris trichura* eggs) to assess the universality of this technology. In summary, this study demonstrates that TiO_2_ can effectively inactivate *H*. *nana* eggs under ultraviolet light irradiation in water, with a superior effect compared to the conventional disinfectant NaClO. The inactivation is achieved by damaging the structural integrity of the eggs and impairing mitochondrial function within the eggs, thereby leading to their destruction.

## 5. Conclusions

**Fig 7:**
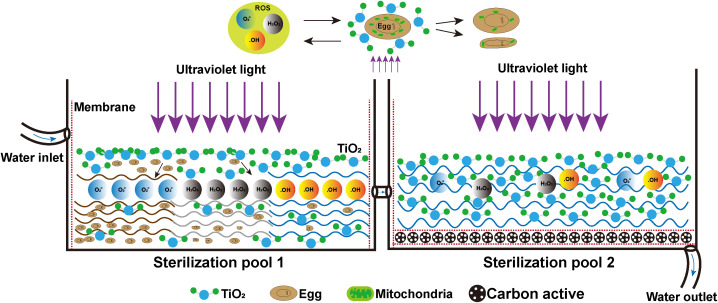
Schematic illustration of the future use of TiO_2_ for the elimination of insect eggs in the water tank of waterworks. Tap water or wastewater from the inlet into the Sterilization pool 1, TiO_2_ in contact with eggs produces ROS when exposed to ultraviolet light, which kills eggs in the water by destroying their structure and the function of the mitochondria within them. The initial sterilization of the water will flow into the Sterilization pool 2, which is further disinfected through the TiO_2_, and at the same time through the activated carbon for filtration, and finally disinfected water discharged through the outlet.

In this study, we found that under optimal conditions (specifically at a concentration of 1.0 mg/mL, light intensity of 0.50 mW/cm^2^, pH 6–8, and exposure to mercury lamp irradiation for 2 h), TiO_2_ photocatalysis effectively inactivated *H*. *nana* eggs. This treatment caused severe structural damage to insect eggs, including egg atrophy and a significant reduction in mitochondrial numbers. It also triggered a substantial increase in intracellular ROS levels and a sharp decline in ATP content. Concurrently, mouse infection experiments confirmed a significant reduction in infectivity. The infection rate in the control group reached 100%, whereas the TiO_2_-treated group showed only a 12.5% infection rate, accompanied by substantial decreases in parasite load and adult worm body length. Moreover, TiO_2_ demonstrated superior efficacy compared to the conventional disinfectant NaClO (0.6%), reducing egg hatching rates below 15%. These results quantitatively confirm that TiO_2_ photocatalysis possesses the ability to kill *H*. *nana* eggs. The findings provide fundamental data and insights for the application of TiO_2_ in combating parasitic eggs in water and offer valuable reference for the future practical use of TiO_2_, paving the way for photoenergy-mediated wastewater and drinkwater decontamination of TiO_2_ for public health benefit (Fig 7).

## Supporting information

S1 Text Supplementary Materials and Methods. (DOCX)

S1 Fig Trypan blue staining for *H*. *nana* eggs. Red arrowheads indicate viable eggs; black arrowheads indicate non-viable eggs; scale bars = 20 μm.
(TIF)

S1 Table Data used for graphing in manuscript. (XLSX)
