# Supplementary material for: TiO2 eliminates Hymenolepis nana eggs via photocatalytic activity
Source: PLoS Negl Trop Dis. 2025 Nov 10;19(11):e0013715. doi: 10.1371/journal.pntd.0013715 (PMC12638027; doi:10.1371/journal.pntd.0013715)
Supplement: S1 Text — (DOCX) [file pntd.0013715.s001.docx]

**Supplementary Materials and Methods**

**1. Optimal NaClO concentration for *H*. *nana* egg hatching**

Firstly, six centrifuge tubes were prepared, each containing 100 μL of *H*. *nana* egg suspension. NaClO solutions of varying concentrations (v/v) (0.2%, 0.4%, 0.6%, 0.8%, and 1.0%) were added to the respective tubes. After thorough mixing, leave the sample at room temperature for 4-6 min. The reactions were then terminated by adding 10 mL of 0.85% (w/v) NaCl solution. The mixture was then centrifuged at 865 x g for 5 min, the supernatant was discarded, and the precipitate was washed twice with 0.85% (w/v) NaCl solution. The final precipitate was resuspended in 200 μL of 0.85% (w/v) NaCl solution. Finally, 100 eggs were counted to calculate the hatching rate under a microscope. Additionally, one drop of the egg suspension was placed on a glass slide, stained with one drop of 0.4% (v/v) trypan blue solution, and 100 oncospheres were counted to assess staining and calculate the oncosphere viability rate.

**2. SEM observation of *H*. *nana* egg damage**

Firstly, a suspension containing 12,500 eggs was taken and treated with PBS, 0.6% (v/v) NaClO, or 1.0 mg/mL TiO_2_ for 2 h or 4 h of photocatalysis with light intensity 0.50 mW/cm², followed by centrifugation at 865 x g for 5 min. The supernatants were discarded, and the precipitate was washed twice with PBS. Secondly, the eggs were then collected by centrifugation and fixed in 2.5% (v/v) electron microscopy fixative at 4°C for preservation. The immobilized egg samples were centrifuged at 865 x g for 5 min, the supernatant was discarded, and the samples were washed three times with ddH_2_O for 10 min each time. Samples were then post-fixed in 1% osmium tetroxide for 1 h, followed by washing three times with ddH_2_O for 10 min each time. Samples underwent graded ethanol dehydration using a concentration series of 30%–50%–70%–80%–90%–95%–100%–100%–100%, with each step lasting 15 min. Then, the dehydrated samples were pipetted onto microporous filter membranes, which were then mounted on SEM stubs using conductive adhesive. The stubs were sputter-coated using an ion sputter coater. Finally, images acquisition was performed using a JSM-IT700HR scanning electron microscope (JEOL, Japan). Regions of interest were selected and imaged to observe the specific pathological changes in the eggs.

**3. TEM observation of *H*. *nana* egg damage**

Firstly, a suspension containing 12,500 eggs was taken and treated with PBS, 0.6% (v/v) NaClO, or 1.0 mg/mL TiO_2_ for 2 h or 4 h of photocatalysis with light intensity 0.50 mW/cm², followed by centrifugation at 865 x g for 5 min. The supernatants were discarded, and the precipitate was washed twice with PBS. The eggs were collected by centrifugation and fixed in 2.5% (v/v) electron microscopy fixative at 4 °C for preservation. Secondly, the fixed egg samples were centrifuged at 865 x g for 5 min, supernatants were discarded and washed twice with 0.1M phosphate buffer (PB). Thirdly, the precipitate was then embedded in 1% (w/v) agarose. The precipitate was carefully transferred into the agarose before solidification to achieve complete embedding. Samples were post-fixed in 1% osmium tetroxide at room temperature in the dark for 2 h. They were then washed three times with 0.1M PB for 15 min each. Samples were dehydrated in ascending concentrations of ethanol 30%–50%–70%–80%–95%–100%–100% for 20 min each time, followed by two rinses in 100% acetone for 15 min each. Fourthly, Samples were soaked in acetone and 812 embedding resin in a 1:1 ratio for 2-4 h at 37°C, then switched to a 1:2 ratio overnight at 37°C. Pure 812 resin infiltration was performed at 37 °C for 5–8 h. The pure resin was poured into embedding molds, and the embedded samples were placed in a 37 °C oven overnight. The embedding molds were then cured in a 60 °C oven for 48 h, and the resulting resin blocks were stored for sectioning. Then, ultra-thin sections (60–80 nm) were cut from the resin blocks using a Leica UC7 ultramicrotome and collected onto 150-mesh copper grids. The grids were stained in the dark with 2% (v/v) uranyl acetate saturated ethanol for 8 min, followed by three washes in 70% ethanol and three in ultrapure water. Then stained with 2.6% (w/v) lead citrate (CO_2_-free) for 8 min and washed three times in ultrapure water. Excess moisture was gently blotted with filter paper, and the grids were left to dry overnight at room temperature in a grid box. Finally, images acquisition was performed using an HT7800/HT770 transmission electron microscope (Hitachi, Japan). Regions of interest were selected to observe and record specific pathological changes in the eggs.

**4. Detection of reactive oxygen species (ROS) and adenosine triphosphate (ATP) in *H*. *nana* eggs**

**4.1 Measurement of intracellular ROS levels**

Firstly, prepare 1 mL of a suspension containing 12,500 eggs and treated with PBS, 0.6% (v/v) NaClO, and 1.0 mg/mL TiO_2_ under photocatalytic conditions for 2 h with light intensity 0.50 mW/cm². Centrifuge at 865 x g for 5 min to collect the eggs and wash twice with 1 mL sterile PBS buffer. Secondly, add 100 μL of diluted 2',7'-dichlorodihydrofluorescein diacetate (DCFH-DA) dye to each group, mix thoroughly with the eggs, and incubate at 37 °C in the dark for 20 min. During incubation, gently invert the samples every 3–5 min to ensure sufficient contact between the probe and the eggs. The samples were washed three times with 1 mL of PBS, centrifuge at 865 x g for 5 min to remove any unbound DCFH-DA. Then, Mean Fluorescence Intensity (MFI) was measured at 485 / 530 nm excitation / emission wavelengths using a SYNERGY-H4 multipurpose microplate reader (Bio-Tek, USA). For fluorescence microscopy: following the above staining procedure, discard the supernatant after centrifugation and resuspend the pellet in 50 μL of pre-chilled PBS. Finally, pipette 10 μL of the suspension onto a glass slide, add antifade mounting medium, and cover with a coverslip. Seal the slide with nail polish and capture images using an upright fluorescence microscope (Eclipse 80i, Nikon Ltd, Japan). All experiments were repeated three times.

**4.2 Measurement of intracellular ATP content in *H*. *nana* eggs**

Firstly, prepare 1 mL of a suspension containing 12,500 eggs and treated with PBS, 0.6% (v/v) NaClO, and 1.0 mg/mL TiO_2_ under photocatalytic conditions for 2 h with light intensity 0.50 mW/cm². Centrifuge at 865 x g for 5 min and discard the supernatant. Add 200 μL of lysis buffer to each pellet and homogenize thoroughly using a tissue homogenizer. Centrifuge at 13,500 x g for 5 min at 4 °C and collect the supernatant for ATP measurement. Secondly, the reagents were thawed on ice at 100 μL of ATP assay workup per sample. Thirdly, the ATP working solution was prepared by mixing the ATP assay reagent with dilution buffer at a ratio of 1:9. The working solution can be temporarily stored at 4 °C. Add 100 μL of the ATP assay working solution to each well. Incubate at room temperature for 3–5 min to eliminate background ATP, thereby reducing baseline luminescence. Then, add 50 μL of the prepared sample to the wells, mix quickly using a pipette, wait at least 2 s, and shake for 5 s. Finally, Relative Light Unit (RLU) was measured at 485 / 530 nm excitation / emission wavelengths using a SYNERGY-H4 multipurpose microplate reader (Bio-Tek, USA).
