# Supplementary figures and images for: TiO2 eliminates Hymenolepis nana eggs via photocatalytic activity
Source: PLoS Negl Trop Dis. 2025 Nov 10;19(11):e0013715. doi: 10.1371/journal.pntd.0013715 (PMC12638027; doi:10.1371/journal.pntd.0013715)

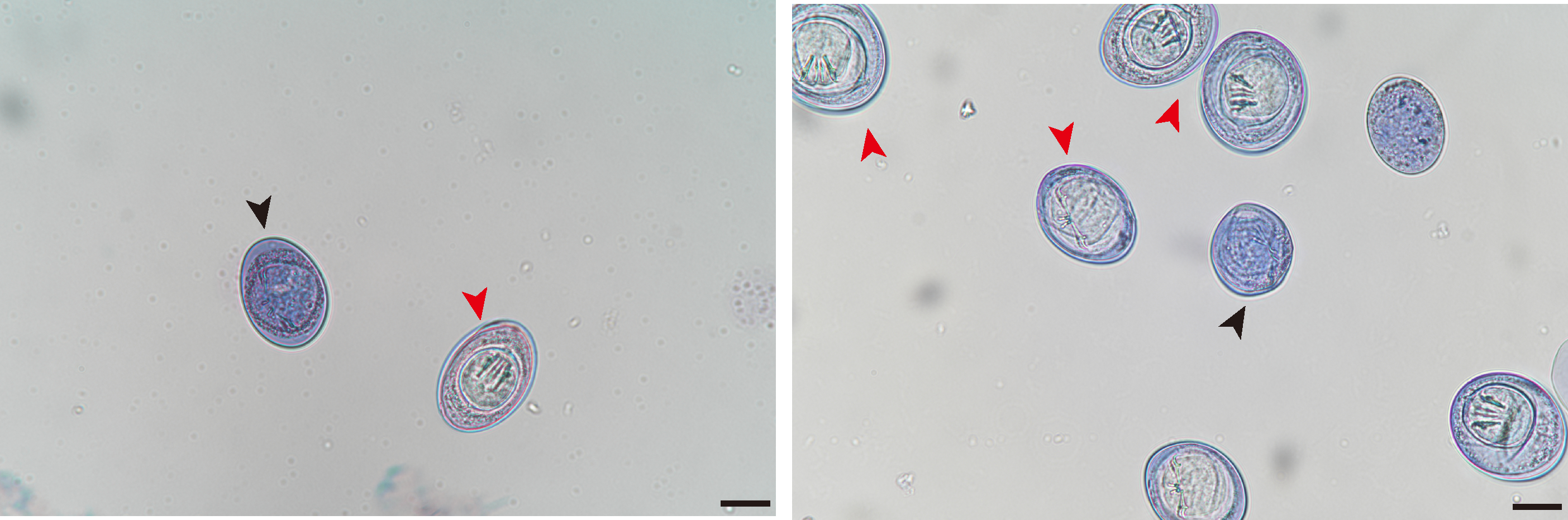

Supplement: S1 Fig — Red arrowheads indicate viable eggs; black arrowheads indicate non-viable eggs; scale bars = 20 μm. (TIF) [file pntd.0013715.s002.tif]
